# Supplementary material for: Ranking of communities in multiplex spatiotemporal models of brain dynamics
Source: Appl Netw Sci. 2022 Mar 14;7(1):15. doi: 10.1007/s41109-022-00454-2 (PMC8921068; doi:10.1007/s41109-022-00454-2)
Supplement: Supplementary file 1 — Additional file 1. Supplementary Information. [file 41109_2022_454_MOESM1_ESM.pdf]

Supplementary Information

1 Parallel Analysis for Dimensionality Reduction

Let X be a $F \times T$ multivariate matrix with F features and T time points. Dimensionality reduction of X by Principal Component Analysis (PCA) requires the selection of the reduced dimension $d < D$. Parallel analysis allows this to be done in a data driven way by comparing the original data set to surrogate data [51]. In parallel analysis, PCA is first performed on X. The resulting eigenvalues can be ordered $\lambda_{(d)}$ so that d is the d largest eigenvalue.

Next, the columns (time points) of X are permuted within each row removing structure from the dataset and this process is repeated $R = 10000$ times producing $X_1, .., X_R$ surrogate data sets with the same row-wise distribution as X. For each X_r we can obtain a corresponding d largest eigenvalue $\hat{\lambda}_{(d),r}$. The optimum choice for d is given by the smallest d satisfying

$$\lambda_{(d+1)} < \hat{\lambda}^{P^{th}}_{(d+1)},$$

where $\hat{\lambda}^{P^{th}}_{(d)}$ is the Pth of the permuted eigenvalues $\hat{\lambda}_{(d),1}, ..., \hat{\lambda}_{(d),R}$. The value of P determines how much the eigenvalues of the components of X must dominate the eigenvalues of the permuted datasets. We choose the percentile $P = 99$. In other words, the first d components in the original dataset must each account for more variance than 99% of the permuted components. This was chosen rather than the standard $P = 95$ in order include as much of the signal in X as possible for HMM model training. The dimensionally reduced data set X^* is thus given by

$$X^* = AX$$

where A is the eigenmatrix of the first d columnwise eigenvectors of X.

2 Directed, Weighted Modularity Score

The directed modularity score $Q(\mathcal{C})$ for a given partition $\mathcal{C} \subset 2^V$ of a weighted, directed graph $G = (V, A)$ with node set V and adjacency matrix A is a measure of how well the partition separates nodes into modules by highly scoring partitions with lower weights on between community edges and higher weights on within community edges. It is calculated as

$$Q(\mathcal{C}) = \frac{1}{m} \sum_{v,v' \in V} \left[A_{v,v'} - \gamma \frac{k_v^{out} k_{v'}^{in}}{m} \right] \delta_{\mathcal{C}}(v, v') \quad (1)$$

$$= \frac{1}{m} \sum_{v,v' \in V} B_{v,v'} \delta_{\mathcal{C}}(v, v'), \quad (2)$$

for $m = \sum_{w,w' \in V} A_{w,w'}$, $k_{v'}^{in} = \sum_{u \in V} A_{u,v'}$, $k_v^{out} = \sum_{u \in V} A_{v,u}$ and $\delta_{\mathcal{C}}(v, v')$ is the Dirac delta function that is one if and only if their exists $C \in \mathcal{C}$ such that $v, v' \in C$ and zero otherwise [55]. The matrix B with elements

$$B_{v,v'} = A_{v,v'} - \gamma \frac{k_v^{out} k_{v'}^{in}}{m},$$

is known as the directed modularity matrix of A [57, 58].

The Louvain implementation we use requires that the modularity matrix B be symmetric. This is achieved by symmetrising, $B' = (B + B^T)/2$ so that

$$\begin{aligned} Q'(\mathcal{C}) &= \frac{1}{m} \sum_{v,v' \in V} B'_{v,v'} \delta_{\mathcal{C}}(v, v') \\ &= \frac{1}{2m} \sum_{v,v' \in V} \left[A_{v,v'} - \gamma \frac{k_v^{out} k_{v'}^{in}}{m} + A_{v',v} - \gamma \frac{k_{v'}^{out} k_v^{in}}{m} \right] \delta_{\mathcal{C}}(v, v'), \\ &= Q(\mathcal{C}), \end{aligned}$$

resulting in the directed modularity score as presented in Equation (2).

3 Measuring the Symmetry of Markov Matrices

It is useful to have a measure to assess the 'degree of symmetry' in a directed network. One way to do this is to consider the energy (as measured by the Frobenius norm) in the weight matrix W which is symmetric [59].

$$\text{Sym}(W) = \frac{1}{4} \frac{||W + W^T||_F^2}{||W||_F^2},$$

where $|| \cdot ||_F$ is the Frobenius norm and $||W + W^T||_F$ is the symmetric part of W. The measure $0 \leq \text{Sym}(W) \leq 1$ is near to 0 when W is 'highly asymmetric' (minimised when W is skew-symmetric i.e. $-W = W^T$) and near to 1 when W is 'highly symmetric' (maximised when $W = W^T$).

4 Community Centrality

The community centrality for the temporal graph $G(P) = (\mathcal{S}, P)$ is calculated using the symmetric undirected version of the transition matrix P to obtain the within community degree centrality z-score, $z(s)$ for $s \in \mathcal{S}$ [61]. Given a partition $\mathcal{U} \subset 2^{\mathcal{S}}$ of the temporal graph into communities, U, this statistic measures how well connected $s \in U$ is in relation to the rest of U. The undirected network is based on $G(P') = (\mathcal{S}, P')$, where $P' = (P + P^T)/2$. The score is

$$z(s) = \frac{\nu_s - \nu_U}{\tau_U},$$

where ν_s is the community-specific degree

$$\nu_s = \sum_{s' \in U - s} P'_{s,s'},$$

and ν_U is the the expected centrality over all other nodes in U,

$$\nu_U = \frac{1}{|U|} \sum_{s \in U} \nu_s.$$

Lastly τ_U is the standard deviation of ν_s for $s \in U$, so

$$\tau_U = \sqrt{\frac{1}{|U|} \sum_{s,s' \in U} (\nu_s - \nu_U)^2}.$$

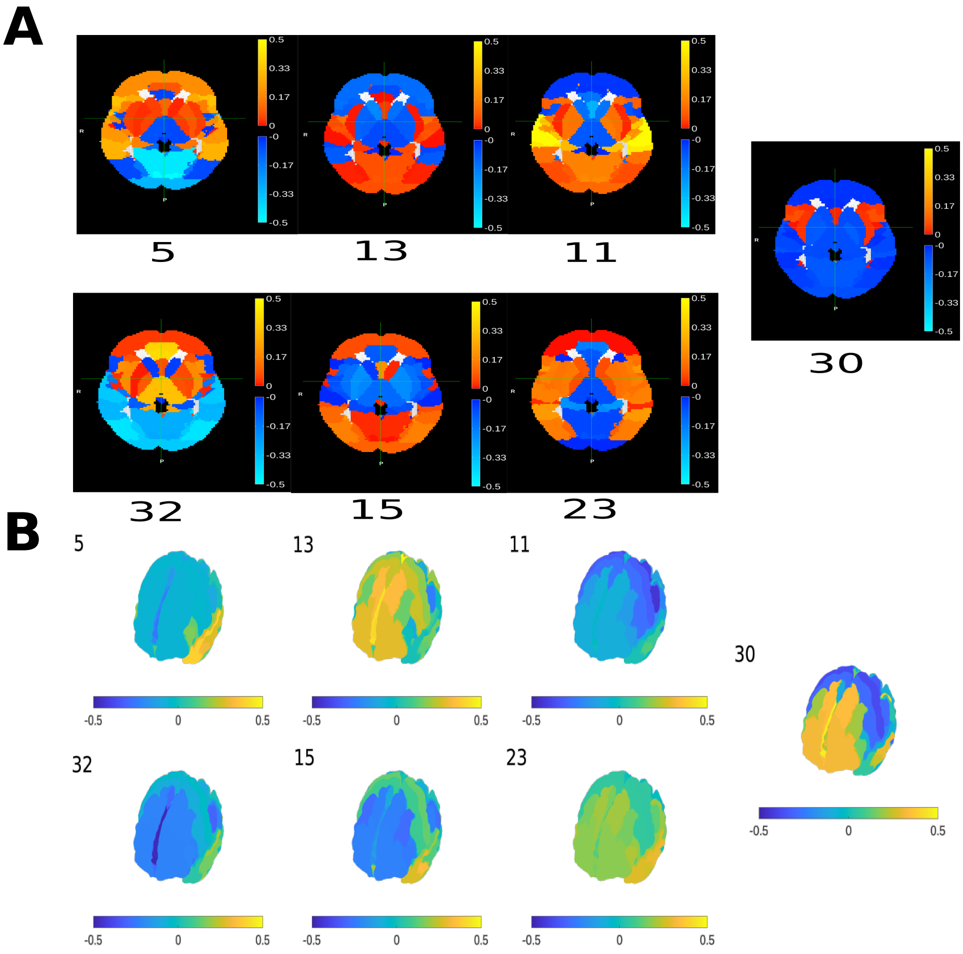

Figure S-1: Hub state mean activity brain maps. (A) The figure shows activity for a central axial slice of the 3D mean activity brain maps of all hub states (the index is determined by the original 33 state HMM). (B) Plots of a surface representation of the same mean activity brain maps for the same hub states.

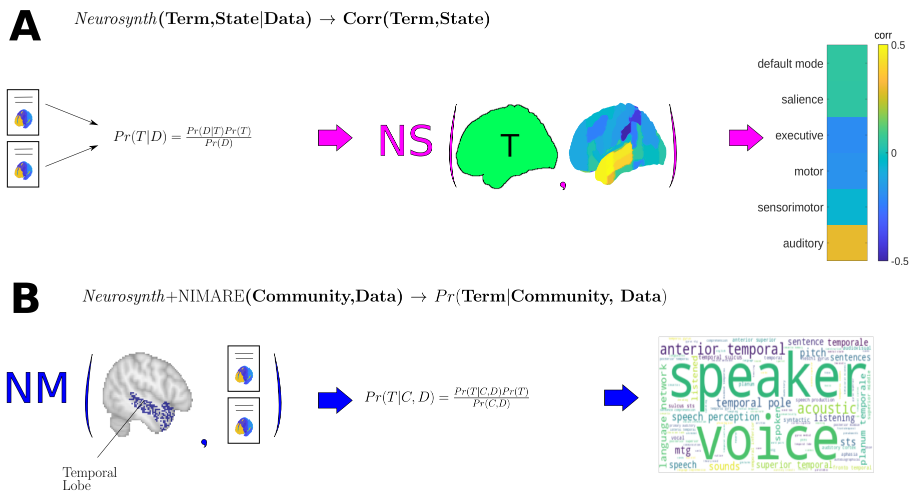

Figure S-2: Diagram overviewing the two *Neurosynth* methods used in our analysis, using the specific example of State 11, Community 1. (A) The first method shows how *Neurosynth* (NS) can be used to generate correlation scores from brain state activity maps and a set of predefined brain terms. In order to perform such a correlation analysis *Neurosynth* requires a corpus of data (D) composed of abstracts with associated brain activation coordinates in (x, y, z) voxel space. We used data from Version 7, dated July 2018 which includes 14,371 studies with 3,178 terms drawn from the data (after removal of numeric characters and words with a frequency less than 1 in 1,000). From this corpus a posterior distribution of term (T) association at each voxel is produced through a Naive Bayes scheme. Term activity maps for a prespecified term are then correlated with the state mean activity brain map and the whole process is repeated for each of the terms of interest. Finally, a full profile of correlations for each term is outputted. (B) For this method we employ the *NiMARE* tool (NM) which uses the *Neurosynth* algorithm to produces a posterior probability over all terms in the corpus for a given selection of voxels. We use as input the voxels defined by the spatial community of brain regions outputted by the community ranking procedure (spatial Community 1 of State 11). The spatial community (C) has been binarised and projected onto the brain map. We show as reference that community activity seems mostly to be located in the temporal lobe, a key region for auditory processing. The final posterior probability of term association is shown as a word cloud where the size of terms is proportional to their predicted probability of association. This method is available in the *NiMARE* Python package.

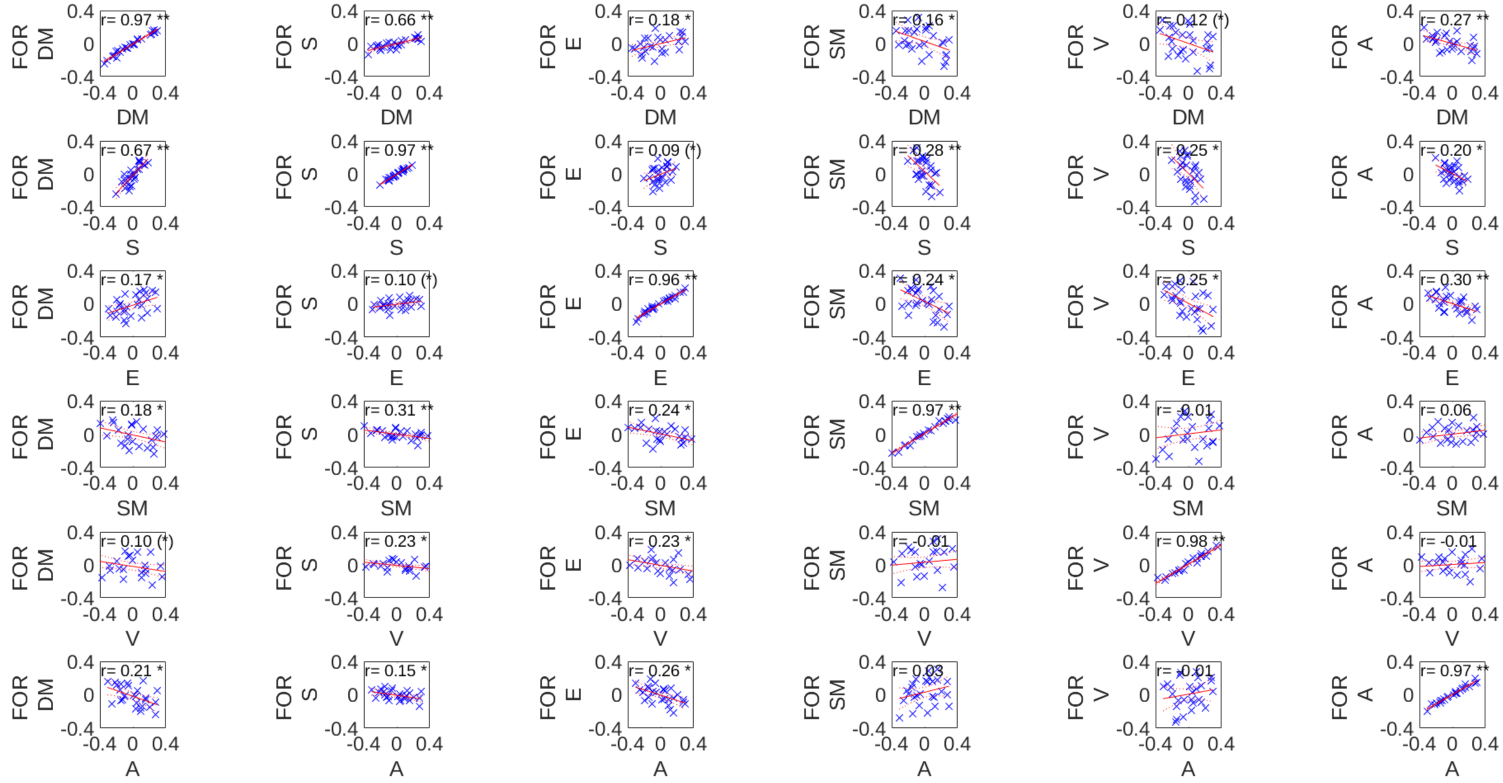

Figure S-3: Grid of scatter plots showing the relationships between *Neurosynth* terms according to their scores for each state using the terms "default mode" (DM), "salience" (S), "executive" (E), "sensorimotor" (SE), "visual" (V) and "auditory" (A) contrasted against the expected score according to the HMM state transition probabilities. Each plot in the grid shows the score for each term associated with a state activity map, plotted against the mean expected score (under the HMM transition probability P) of the next forward (FOR) timestep. The comparison between state score and expected score forward in time demonstrates spatiotemporal relationships between the states (layers) of the network.

5 Validation with Synthetic Data

This section details the validation of key features of the HMGM selection and analysis framework using synthetic (simulated data). Where possible, similar simulation approaches are used in order to make the simulated pipeline as coherent and true to the data as possible while taking into account computational and practical constraints.

5.1 Parallel Analysis Experiments

We use PCA to reduce the computational complexity and noise in the modelling process. In order to determine whether Parallel Analysis (see Section 1) could be used to obtain reasonable and consistent estimates of the embedding dimension (number of PCs) even when variability is high, we performed experiments on synthetic data simulating functional activity from $D = 63$ brain regions (the same dimensionality as the real data). Brain activity is simulated from a state $s \in \mathcal{S}$ with multivariate normal observation assumed to have state mean activity $\mu(s)$ and noisy covariance matrix $\Sigma(s)$. These number of states is the same as the observed $K = 27$ state model.

In this simulation, communities in state s are embedded into a noisy covariance matrix, $\Sigma(s)$, as cliques with correlation r (this differs from the variable relationships between brain regions in the same community seen in the observed model). Each community, C, is a member of partition $\mathcal{C}_s$. These brain region communities have identical membership to the actual communities in the observed model. This helps to provide clique communities of variable sizes that are consistent with the observed model. In order to account for community structure as well as noise and intersubject variability, the state covariance matrix is generated from an Inverse Wishart distribution with scale matrix $\Psi(s, r)$,

$$\Psi(s,r)_{i,j} = \begin{cases} 1 & \text{if } i = j \\ r & \text{if } \delta_C(i,j) \\ 0 & \text{otherwise.} \end{cases},$$

and degrees of freedom $\nu \geq D - 1$ which represents the amount of variability in $\Sigma(s)$. The degrees of freedom are low when reflecting noisy data with a lot of inter- and intrasubject variability and high when there is assumed to be little noise in $\Sigma(s)$. We explore the specific case $r > 0$ for parametric simplicity. In practice, the particular realisation of the state intracommunity correlation may be positive or negative.

The simulation of dynamic brain activity for a given number of degrees of freedom (ν) and intracommunity correlation r works as follows:

1. The state covariance $\Sigma(s)$ matrices are generated from an $IW(\Psi(s, r), \nu)$ distribution with degrees of freedom ν, community membership com-

ing from Louvain community detection as performed on the observed model (with $\gamma = 2$), and intracommunity correlation r. In addition, state mean activity $\mu(s)$ is generated using a multivariate normal distribution with zero mean and unit variance.

2. Then $n = 20$ samples are generated from the state.

3. Step 1 and 2 are repeated $t = 100$ times to generate a sample of size $N = 2000$ which is then mean subtracted and divided by the standard deviation for each dimension respectively to produce a dataset X with zero mean and unit variance.

4. Parallel Analysis is performed on the sample X.

5. This process is repeated $R = 10$ times for a particular parametrisation to generate an empirical distribution of PCs suggested by parallel analysis.

The result of generating samples for different values of r (degree of correlation between regions) is shown in Figure S-4 over a range of noise in the state covariance matrix (represented by different ν). When the noise in state covariance is high (i.e. ν is high) then the number of recommended dimensions is low (suggesting little signal above noise in the data), however as the noise decreases the number of PCs recommended by Parallel Analysis increases to capture more of the signal. The method behaves similarly no matter the intracommunity correlation strength, although for higher values of r the number of PCs required at low noise decreases slightly. This could reflect that the high level of correlation results in fewer PCs needed to represent the data.

5.2 Clique Community Recovery Experiments

In order to determine whether state Markov Information matrices (see Equation (3) of the main text) could reasonably be used to recover the spatial community structure of a state, we tested this method on a clique community recovery task in which clique communities are embedded in a noisy covariance matrix generated as in Section 5.1. We then compare the performance to a simple undirected method using absolute correlation to generate an graph from a covariance matrix. We also examine the effects of model parameters on clique recovery performance, notably the degrees of freedom ν, the within-clique correlation r and the number of principal components used to reduce the matrix dimension (as in Section 2.1 of the main text).

Community detection algorithm performance was computed using the Adjusted Rand Index (ARI) [64], which measures the similarity between the true partition $\mathcal{C}$ and that calculated by the Louvain algorithm. A moderate

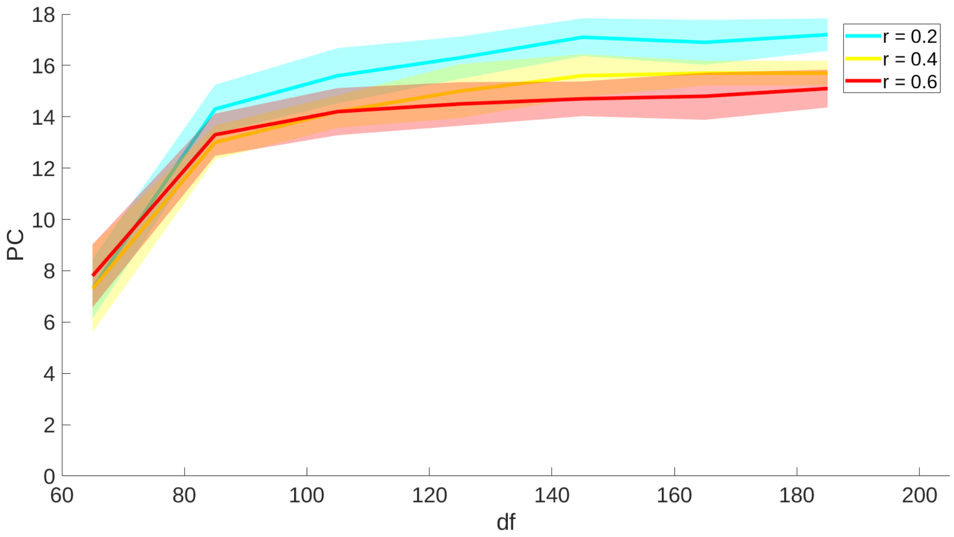

Figure S-4: This figure shows the number of Principal Components (PCs) recommended by the Parallel Analysis (PA) method for a small data set of synthetic brain activity data. The colours show the level of intracommunity correlation within the synthetic brain state (based on actual observed community membership). The shading shows the standard error in PCs while the colour indicates the intracommunity correlation in activity.

ARI score is 0.6 or above.

In our model preprocessing procedure, data is first dimensionally reduced to reduce noise and complexity. We thus perform the same transformation on the sampled covariance matrix as in Equation 2 of the main text. In order to approximate real partitioning, true partitions were sampled from the set of $K = 27$ partitions determined from data in the main text and 1000 $D \times D$ matrix realisations of the inverse Wishart distribution were generated for each setting of the simulation parameters: degrees of freedom, PCs and within-clique correlation (r). Figure S-5 shows the result of these experiments for both methods. The results show that for moderate levels of within-clique correlation, both graph construction methods are able to recover true community activity for a reasonably large range of parameters. Notably, when the number of principal components is either too high (including too much noise) or too low (removing too much signal), community detection performance suffers, underscoring the importance of reasonably choosing this parameter.

The results suggests that the optimum number of PCs to recover the community structure embedded in a covariance matrix (depending somewhat on the amount of noise) is between 10 and 20. This is in agreement with the

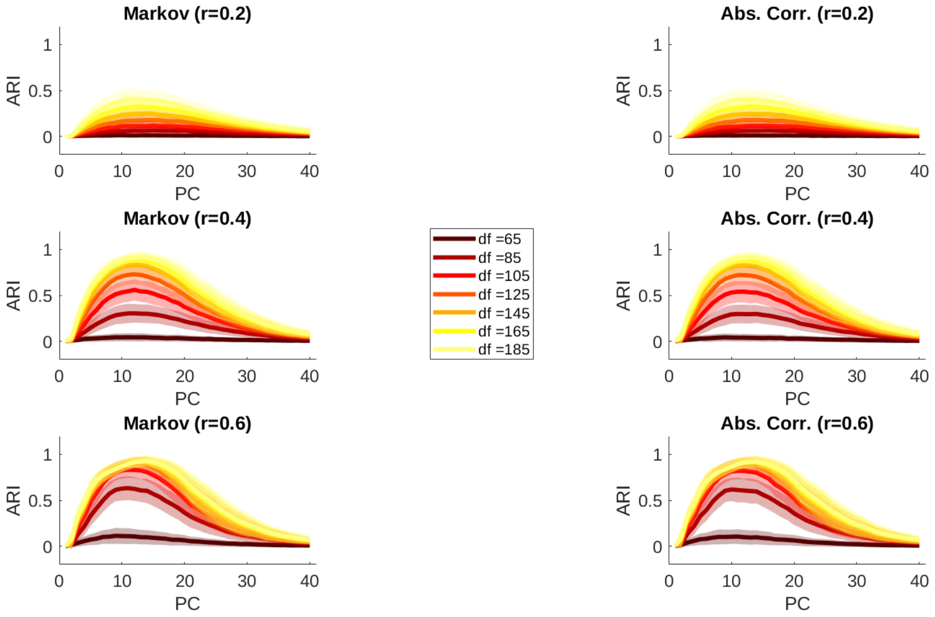

Figure S-5: This figure compares the performance of the Louvain community detection algorithm on clique-recovery tasks for two different methods of constructing graph models from simulated state covariance matrices of size $D = 63$. The first method is the directed Markov Information matrix method (left) and the second is the method of undirected graph construction by absolute correlation (right). ARI scores for a fixed number of degrees of freedom (df), denoted ν, are plotted across a variable number of principal components (linear embedding dimension), with higher ν corresponding to reduced noise in the covariance matrix. Performance is almost identical for both methods with performance improving as either the clique strength or degrees of freedom increases. The relationship between linear embedding dimension (PC) and model performance is not monotonic but rather, improves as more signal-rich components are included, deteriorating when higher noisy components are included.

results of Parallel Analysis simulation (see Figure 1) as even when noise is relatively high ($\nu \geq 105$), the number of recommended PCs is above 12. If the HMM training procedure is able to recover a moderately accurate representation of the covariance matrix (with some allowance for noise), and the within community correlation is strong ($r \geq 0.4$), our simulation indicates that parallel analysis is able to recover the community structure with relative accuracy (having a moderate ARI).

5.3 Hidden Markov Model Selection Experiments

Finally, we test the ability of the cross validated maximum entropy model selection procedure to select the correct number of HMM states from synthetic data that has already been dimensionally reduced to $d = 9$ dimensions. We apply the same additional constraint as in Section 3.2, that the state must be present in at least 25% of subjects to be included. In addition to testing whether the selection criterion can identify the correct number of states, we also test whether the model identified and trained on this data can recover the temporal community structure of the states.

Data from $N = 15$ subjects was generated over $T = 200$ time points. The data was generated from an HMM with $K = 6$ states and d MVN observations that includes subject-specific noise in the covariance matrix. The number of states is considerably less than for the observed model but this was done due to computational constraints on simulating and running multiple models.

Each state covariance matrix is assumed $\Sigma(s) \sim IW(\mathbf{I}, 40)$ distributed (chosen to be roughly $\nu \approx d + 30$ for reasonable hypothetical recovery if community structure were present), where $\mathbf{I}$ is the identity matrix, with mean $\mu(s) \sim MVN(\mathbf{0}, \mathbf{I})$. The state transition matrix $P(c)$ was chosen so that given correctly selected $c > 0$ the matrix can be potentially partitioned into two communities, a strongly connected community and a weakly connected community, each with equal membership, so that $P(c)$ equals

$$\left(\begin{array}{ccc|ccc} 0.97-2c & c & c & 0.01 & 0.01 & 0.01 \\ c & 0.97-2c & c & 0.01 & 0.01 & 0.01 \\ c & c & 0.97-2c & 0.01 & 0.01 & 0.01 \\ \hline 0.01 & 0.01 & 0.01 & 0.87-2c & c+0.05 & c+0.05 \\ 0.01 & 0.01 & 0.01 & c+0.05 & 0.87-2c & c+0.05 \\ 0.01 & 0.01 & 0.01 & c+0.05 & c+0.05 & 0.87-2c \end{array}\right),$$

where the lines indicate the two temporal communities with the first being the weaker community. As in the observed model for wakefulness, the probability of self-transition is high in general with a relatively low probability of leaving a given community of 10^{-2}.

In order to incorporate subject specific differences in state activity, observations for subject i are sampled from a slightly perturbed model with $\Sigma(s, i) = \Sigma(s) + \epsilon\hat{\Sigma}$ where $\hat{\Sigma}$ is sampled from the same distribution as $\Sigma(s)$ and similarly $\mu(s, i) = \mu(s) + \epsilon\hat{\mu}$ where $\hat{\mu}$ is sampled from the same distribution as $\mu(s)$. Here we choose $\epsilon = 0.01$.

Figure S-6A shows the results of model selection and HMM fitting when the temporal community coupling parameter is set to $c = 0.05$. As in the observed model (see Section 3.2), there is a clear relationship between cross-validated log-likelihood maximisation and entropy maximisation. The neg-

ative cross-validated entropy appears to decrease near monotonically with the number of states, as does the likelihood. The resulting minimum entropy model has $K = 14$ initial states, 8 of these were removed due to not being present in enough subjects and providing a final model with 6 states as expected. It is also clear from Figure S-6B that the method recovers the underlying community structure of the network even when the preference for intracommunity transition over intercommunity transition is relatively weak. The difference in intracommunity transition probability are also evident from the trained model. Similar results are seen when $c = 0.15$, in Figure S-6C. In this case initially $K = 12$, but pruning non-general states again gives 6 states in the final model with the expected community structure. The fact that the number of states is overestimated in both cases shows the importance of the pruning step in determining the final model. In addition, the optimal resolution, γ, according to the Variation of Information was found to be the same for both models with $\gamma = 0.08$.

In both models it is clear that the entropy increases with additional states but the increases begin to slow down after the true number of states is exceeded. It thus may be beneficial to consider not only the absolute global minimum but also the first local minimum when assessing potentially viable models for exploration.

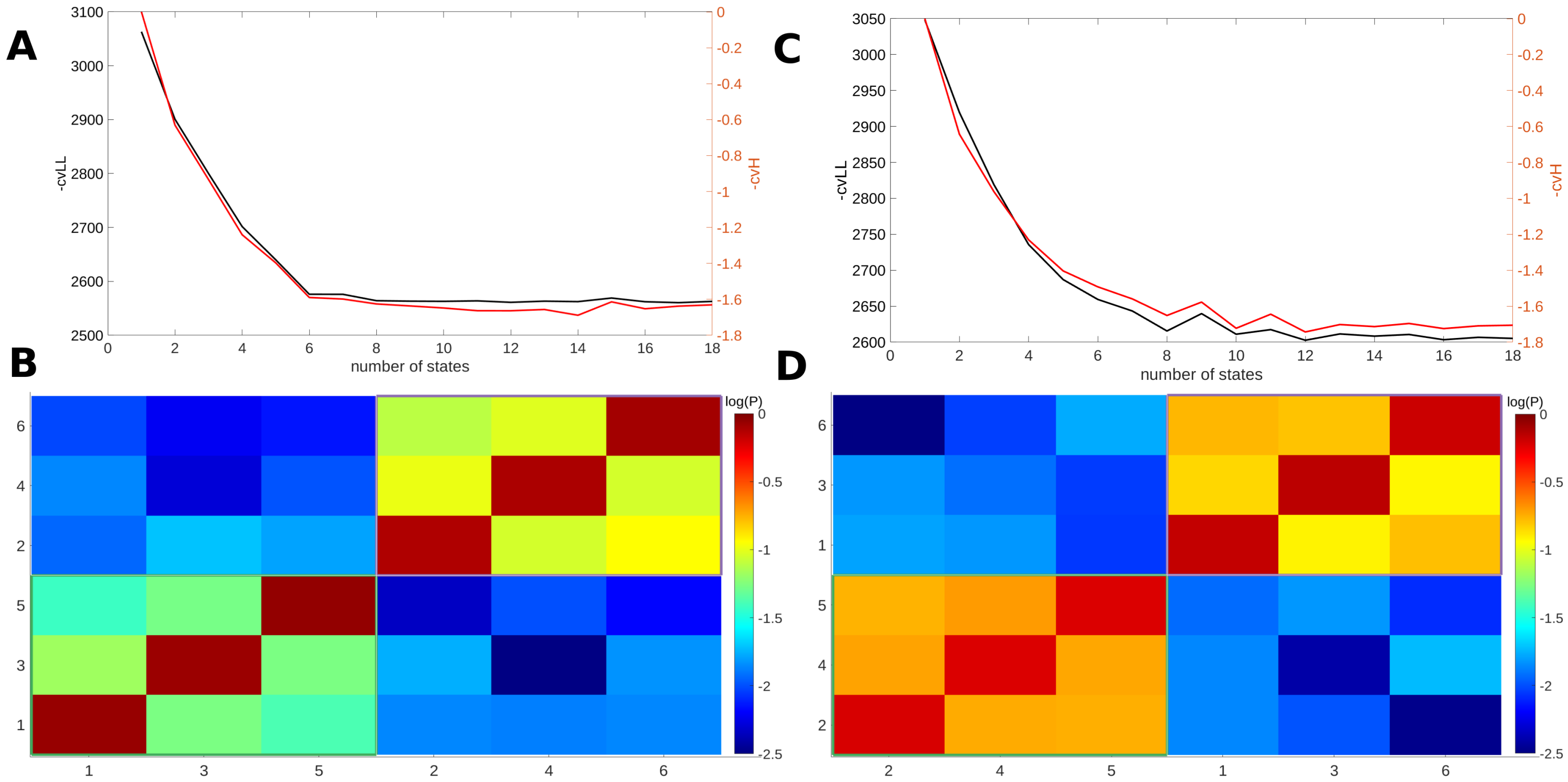

Figure S-6: Model selection and temporal community detection based on synthetic data simulated from $N = 15$ subjects with subject-specific noise. (A) Shows the results of model selection using the cross-validated log-likelihood (black, left axis) and cross-validated entropy (red, right axis). (B) Shows the log-transition matrix (colorbar with log-probability of transition) for the $c = 0.05$ model with lines dividing the two communities detected using the Louvain method with $\gamma = 0.08$ (determined by minimum Variation of Information). The axis labels are the states. The exact state label is arbitrary and so should not be expected to match the original numerical labels. (C) This figure shows the model selection results for for the higher intracommunity edge strength model with $c = 0.15$. (D) Shows the temporal community detection results for the final model with again $\gamma = 0.08$. In both cases it is clear that the method recovers the correct community structure.
